# Valvular heart diseases in women

**DOI:** 10.1186/s43044-021-00184-3

**Published:** 2021-06-26

**Authors:** Ghada Youssef

**Affiliations:** Cardiology Department, Kasr Al Ainy Hospitals, Cairo University, Cairo, Egypt

**Keywords:** Women, Valvular heart diseases, Pregnancy

## Abstract

**Background:**

Valvular heart disease is a common health problem affecting both sexes and all age groups. Almost all guidelines are based on studies that mainly involved male patients.

**Main body:**

The features of valvular heart diseases in women were essentially derived from small observational studies. These studies have shown that women differ from men in terms of the type of valve disease, pathology affecting the valve, perception of symptoms, parameters measured in echocardiography, response to drugs, surgical techniques, and postoperative outcomes. This review was conducted to demonstrate the uniqueness of valvular heart diseases in women and raise awareness about the need for sex-based randomized study designs to provide a piece of proper evidence to support suitable sex-based guidelines and recommendations. Moreover, it briefly describes the effects of pregnancy on women with valvular heart diseases and the impact of the latter on the course of pregnancy and the well-being of the mother and fetus.

**Conclusion:**

Valvular heart diseases in women are different from those in men. Sex-based guidelines for managing valvular heart diseases are needed.

## Background

Is it true that men are from Mars and women are from Venus, as the famous book by Mr. John Gray implies? Although the book focused only on the psychosocial differences, yet in fact, several biological and socio-behavioral differences exist between men and women. Cardiac anatomy and physiology are quite different in women. In addition, the unique hormonal-derived physiological changes that occur during menstruation, pregnancy, and menopause make women even more unique. These distinct physiological features can explain the different pathological characteristics in women, with different prevalence and prognoses of diseases.

Women with valvular heart diseases have certain characteristics that are quite different from those of men. Women differed in terms of prevalence, clinical presentation (perception of symptoms), sensitivity and specificity to investigational procedures (different echocardiography normal cutoff values), response to drugs (different prescribed doses as well), management plans (mainly physician-related), and valve disease outcomes [1]. Thus, diagnosis and treatment of valvular heart diseases should be tailored to the respective sex and body size. However, the guidelines still lack evidence that defines these sex-related differences in the context of valvular heart diseases. This review was conducted to shed light on the unattended section of valvular heart diseases in women and raise awareness about these differences. Because little is known about the mechanism by which valvular heart diseases are affecting women differently, this review was conducted to also invite researchers to design studies that answer the still mysterious, not fully understood clinical points of these differences.

## Main text

### Pathophysiology

Women and men with the same degree of valvular lesions usually behave differently. This is because of the inherent cardiovascular sex-related anatomical and physiological differences, which are aggravated when significant hemodynamic changes occur, as in cases of significant valvular lesions.

Women have smaller heart sizes, which may be attributed to estrogen, which may explain why the hearts of post-menopausal women grow faster than those of men after the age of 70. Women have lower left ventricular (LV) end-diastolic volumes and pressures and higher ventricular ejection than men, and the hearts of women are more distensible and hyperdynamic than those of men. In addition, women have a lower oxygen-carrying capacity (owing to the lower hemoglobin (Hb) level and blood volume), higher cardiac output during exercise, and higher heart rate response to submaximal exercise than men [2].

Throughout their life, women go through fluctuating levels of sex hormones. The monthly hormonal changes related to the menstrual cycle, the hormonal changes during pregnancy, and the decreased levels of sex hormones after menopause could affect the cardiovascular hemodynamics and blood physiology, which could affect the clinical status of patients with valvular lesions. Platelet count, blood viscosity, and hematocrit decrease during menstruation. In addition, heart rate and systolic blood pressure show different patterns according to the menstrual phase [3].

Alternatively, pregnancy causes hyperdynamic circulation, and the cardiovascular changes during pregnancy may be deleterious to patients with significant valvular lesions. For example, the increased blood volume, heart rate, and cardiac output during pregnancy may precipitate heart failure in patients with severe mitral stenosis (MS) [4]. Pregnancy can unmask an undiagnosed valvular lesion or may precipitate symptoms in a previously asymptomatic patient. In addition, pregnancy causes a state of hypercoagulability, which may increase the incidence of thromboembolic events in patients with valvular atrial fibrillation (AF) and mechanical heart valve prosthesis [5].

The decrease in estrogen after menopause has many unfavorable effects on the cardiovascular system. Menopause is associated with increased blood pressure, increased sympathetic tone, abnormal lipid metabolism, android fat distribution, and increased deposition of collagen and elastin in the heart. These changes may accelerate the aortic and/or mitral valve (MV) (and mitral annulus) degeneration and calcification. Because designing a model on this changing hormonal background is difficult, women are usually under-represented in heart valve-related clinical trials [6].

### Prevalence and etiology of valvular lesions

Women have a higher prevalence of MV prolapse, with a more diffuse myxomatous MV disease and extensive leaflet thickening, than men [7]. MV prolapse is often diagnosed in young women, and the incidence decreases with age [8]. Women have a higher prevalence of MV stenosis (with 3:1 women predominance), either rheumatic [9] or degenerative (where women develop mitral annular calcification more than men) [10]; rheumatic mitral regurgitation [11]; tricuspid valve stenosis; and secondary tricuspid valve regurgitation [12].

The prevalence of a bicuspid aortic valve (AV) disease in men is three times higher than that in women, and on presentation, women present mainly with moderate/severe aortic stenosis (AS), whereas men present mainly with moderate/severe aortic regurgitation. Women with a bicuspid AV have a smaller aortic annulus, a smaller LV outflow tract, and a more hypertrophied LV [13].

Approximately three-fourths of patients with congenital AS and aortic regurgitation are males [14], whereas in congenital pulmonary stenosis, the male-to-female ratio is 1:1 [15].

Women with degenerative AS have less valvular calcification and a more pronounced valve fibrosis, and the LV generates higher gradients and contracts more forcibly than men [16].

### Diagnosis

To properly determine the severity of a valvular disease, the treating physician should check for certain red flags. Symptoms, cardiac dysfunction, and the enlargement of the cardiac chambers are considered keystones for the decision of valve surgery in patients with valvular heart diseases.

A recent study has shown that the normal cardiac size is affected by four factors: age, sex, weight, and height [17]. These factors are well-appreciated in pediatric patients, with different curves for boys and girls, different ages, and body surface area (BSA). However, in adults, defining different values for different individuals is not a common practice, particularly values according to the patient’s sex.

Before sending patients to valvular surgery, determining the cardiac size is crucial. As these values are quite different in women compared with those in men; thus, guidelines should provide different cutoff values for both sexes in case a heart valve surgery is considered.

### Perception of symptoms

As far as we know, no specific studies have focused on sex-related differences in the perception of symptoms due to valvular diseases. Patients with valvular heart diseases usually complain of heart failure symptoms, and valvular disease is considered the most common cause of heart failure in women [18]. The data on how women perceive heart failure symptoms differently were mainly derived from the SOLVD trial, which has shown that women complain of shortness of breath more than men and had a higher incidence of thromboembolic events with low LV ejection fraction than men [19]. Women with heart failure are characteristically more susceptible to arrhythmias, especially AF [18].

Women with mitral regurgitation mainly complain of shortness of breath [11], whereas women with low-gradient, low-ejection fraction AS mainly complain of syncope [1].

It was observed that women who were sent for surgical AV replacement for degenerative AS had a more pronounced exertional dyspnea and higher frailty score than men [20, 21].

Despite the lack of studies focusing on the analysis of chest pain symptom in patients with valvular heart diseases (as in patients with severe AS or severe aortic regurgitation), it is generally known that women with ischemic chest pain usually describe atypical anginal pain as pressure or dull sensation in the chest, and they usually locate pain in more than one region across the chest wall [22].

The difference between men and women is not only biological but also social, cultural, behavioral, and psychological. That is why the approach of physicians to female patients should be different from their approach to male patients, and they should consider all aforementioned possibilities when interpreting the symptoms of a patient.

### Echocardiographic diagnosis

The American Society of Echocardiography in its recent guidelines has demonstrated that the normal cardiac dimensions and functions should be indexed to the BSA, and they have defined different values for women and men (e.g., the normal LV ejection fraction is defined as more than 52% in men and more than 54% in women) [23]. In the NORRE study, which has included 414 women and 320 men, the LV dimensions, volumes, and mass were larger in men than in women, even after normalization for the BSA, whereas the LV ejection fraction was significantly higher in women than in men [24]. However, in the ESC guidelines of valvular heart diseases, women were mainly mentioned in the section of pregnancy, and no sex-specific cutoff values for surgery were defined in their recommendations [25].

The echocardiographic cutoff values indicating valve surgery were mainly derived from male patients who inherently have bigger hearts. Applying these cutoff values on women, who have lower body sizes and smaller hearts, may explain why women are presented at an advanced stage of valve disease, as they take longer to reach the respective cutoff values for valvular surgery [26]. Thus, using indexed cutoff values is preferable to overcome this problem in women and in patients with small BSA.

### Management

#### Drugs

Patients with valvular heart diseases are usually prescribed heart failure drugs. One of these drugs is digoxin, which is used to control the heart rate in patients with AF and systolic dysfunction. Digoxin is associated with a higher all-cause mortality in women with heart failure and reduced ejection fraction (< 45%). In addition, women are more likely to have a high median serum digoxin level, and this may explain the higher incidence of digitalis toxicity in women [27]. Data derived from heart failure studies have revealed that other commonly used drugs, such as beta-blockers (e.g., for patients with MS), angiotensin-converting enzyme (ACE) inhibitors (e.g., for patients with dilated LV), and diuretics (for symptomatic volume overload), were equally effective in women and men [28, 29]. It is worth noting that ACE inhibitors and angiotensin receptor blockers (ARBs) are absolutely contraindicated during pregnancy and lactation because of the risk of fetopathy and neonatal renal impairment [30].

#### Percutaneous intervention

Balloon dilatation of rheumatic MS is the recommended treatment option when MS is symptomatic, severe, with pliable non-calcified leaflets, and without significant mitral regurgitation or left atrial thrombi. Evidence proves that women have better outcomes of percutaneous balloon mitral valvuloplasty [31]. In patients with degenerative MS, calcification mainly affects the base of the leaflets with no commissural fusion, making percutaneous/surgical commissurotomy technically non-feasible [1].

Transcatheter AV replacement (TAVR) is a recently introduced treatment option for patients with a high surgical risk that precludes the option of surgical AV replacement. Women undergoing TAVR are older, have a lower prevalence of coronary artery disease, and have a better LV function than men. Yet, the incidence of periprocedural coronary obstruction, the rate of vascular complications, and the need to convert to open surgery are higher in women than those in men [32].

#### Valve surgery

##### AV surgery

Performing AV surgery in women is technically demanding. Women’s small body size requires the use of smaller valve prostheses and pre-replacement annular dilatation [33]. Women with degenerative AS who underwent surgical AV replacement were older than men [20, 21], and this observed age difference is probably due to the late presentation to physicians (as women tend to underreport symptoms) and late referral to surgery (with more advanced disease state and more eccentric LV remodeling) [20].

Women have a higher incidence of late stroke after AV mechanical prosthesis surgery [34], and the results from the OBSERVANT registry have shown that the postoperative short-term survival was lower in women, but the long-term survival in women was comparable to that in men [21].

##### MV surgery

Primary MV disease repair improves survival in patients with mitral regurgitation, irrespective of sex. In secondary MV disease, women and men showed comparable results regarding clinical outcomes and LV reverse remodeling with a minimally invasive MV repair [1].

Women with degenerative MS are characteristically old with many comorbidities, and thus, they have considerable perioperative risk [1].

### Infective endocarditis in women

Infective endocarditis is more common in men than in women (with a ratio of 2:1 in men and 9:1 in women). A recent study that explored the sex-related differences in patients with infective endocarditis has shown that women are characteristically younger, are more predisposed to cardiac lesions (rheumatic mainly), and suffer more complications than men. Alternatively, men with infective endocarditis mostly were intravenous drug abusers on a background of a structurally normal heart [35].

### Valvular heart diseases during pregnancy

Pregnancy is a uniquely challenging situation in a woman’s life. It causes a significant burden over the circulatory system, especially in women with significant valvular lesions. Because of the expected health hazards to the mother and/or fetus, a preconception counseling is recommended, and any significant valvular lesion should be corrected before contemplating pregnancy. Maternal and/or fetal complications may develop secondary to the valve disease itself, drugs used to control heart failure, anticoagulation used for AF or for the prosthetic heart valve, or the interventional procedures that may be needed during pregnancy [4].

Severe MS is the least tolerated valve lesion during pregnancy (mWHO-IV), and thus, correcting the lesion before conception is recommended. Women with severe MS who present late during pregnancy can be offered percutaneous/surgical correction of the MV during the second trimester [4].

Asymptomatic severe AS is fairly tolerated during pregnancy (mWHO-III), whereas symptomatic AS is poorly tolerated (mWHO-IV), and pregnancy should be discouraged until the lesion is corrected.

Valvular regurgitant lesions are well tolerated during pregnancy, as long as the LV function is maintained [4].

Women with prosthetic cardiac valves are considered mWHO-III risk category and should be closely followed up by the Pregnancy Heart Team throughout pregnancy and during the early postpartum period (6 weeks postpartum). Anticoagulation for these patients carries several issues that have not yet been completely understood, including the proper anticoagulant drug to use during pregnancy, the proper dose of the selected drug, the timing of switching between drugs, and how to avoid complications. Recent European and American guidelines have provided algorithms for anticoagulation in pregnant women with prosthetic heart valves, and they have proposed two algorithms according to the vitamin K antagonist (VKA) doses needed to control the international normalized ratio to the desired level, in the first trimester. These algorithms seem helpful in making a decision about anticoagulation, but several issues related to these algorithms remain unanswered. First, they did not define the recommendation in patients who take the exact cutoff dose (i.e., 5-mg warfarin, 3-mg phenprocoumon, and 2-mg acenocoumarol). Second, the recommendations only attempted to avoid the risks of high VKA doses in the first trimester (for fear of embryopathy), but they did not define the anticipated complications (fetal internal hemorrhage and hydrocephalus) in patients who require high VKA doses (e.g., warfarin > 10 mg/day) in the second and third trimesters until switching to heparin at the 36th week of gestation. Third, the finding that all recommendations of anticoagulation during the first trimester were class II recommendations, and this is probably because the evidence supporting class I recommendations is limited [4].

Because of the aforementioned issues related to anticoagulation in patients with mechanical prosthetic valves, the guidelines recommended that whenever a valve replacement surgery is indicated in women at the child-bearing age, it is preferable (class IIa, level of evidence C) to consider a bioprosthesis valve to avoid maternal and fetal hazards associated with anticoagulation [25].

Pregnancy may, in part, explain why young women are under-represented in clinical trials because of the potential consequences of the study protocol on maternal and/or fetal well-being and the potential associated legal issues.

#### Valvular heart disease in Egypt

Egypt is one of the countries where rheumatic fever is endemic and rheumatic heart diseases are considered important causes of morbidity and mortality among young Egyptians. Valvular heart disease is the top 2nd cause (after ischemic heart disease) of heart failure, and this was observed across different regions of Egypt, including Cairo, Alexandria, and especially Upper Egypt, where valvular heart disease accounted for a quarter of heart failure cases [36]. El Aroussy et al. have performed a study on 48,930 school children and found that the prevalence of rheumatic valvular heart disease was 0.07% among school children aged 6–18 years [37]. Another study involving more than 17,000 Egyptian individuals has shown that the prevalence of rheumatic valvular heart disease was higher among females, residents of rural areas, and patients with low socioeconomic status. Female sex was an independent predictor of developing rheumatic valvular heart disease in a multivariate analysis with a relative risk of 1.2 compared with males [38]. Since the middle of the last century, the prevalence of rheumatic heart disease has declined in Egypt because of the improved healthcare services, the national implementation of rheumatic heart disease prevention programs, and the improved socioeconomic standard and quality of life [39]. Unfortunately, no available Egyptian data exist regarding sex-related differences in valvular heart diseases.

## Conclusion

Valvular heart diseases have characteristic features in women, including different prevalence, valve pathology, clinical picture, and disease outcomes. Special management recommendations exist for pregnant women with valvular lesions, and a special team (the Pregnancy Heart Team) should be responsible for their counseling, follow-up, and decision making. Women are under-represented in valvular clinical trials, and evidence to support sex-based recommendations remains limited. Future studies should focus on tailoring diagnosis and management for valvular heart diseases according to the patients’ sex and body size.

## Data Availability

Not applicable.

## Abbreviations

ACE: Angiotensin-converting enzyme
AF: Atrial fibrillation
ARBs: Angiotensin receptor blocker
AV: Aortic valve
BSA: Body surface area
EF: Ejection fraction
ESC: European Society of Cardiology
LV: Left ventricle
MS: Mitral stenosis
MV: Mitral valve
NORRE: Normal Reference Range for Echocardiography
SOLVD: Study of Left Ventricular Dysfunction
TAVR: Transcatheter aortic valve replacement
VKA: Vitamin K antagonists
WHO: World Health Organization
